# Allopurinol-Induced Granulomatous Hepatitis: A Case Report and Review of Literature

**DOI:** 10.1177/2324709617728302

**Published:** 2017-09-08

**Authors:** Umair Iqbal, Hafiz Umair Siddiqui, Hafsa Anwar, Ahmad Chaudhary, Abdulhadi Affan Quadri

**Affiliations:** 1 Bassett Medical Center, Cooperstown, NY, USA; 2 Cleveland Clinic Foundation, Cleveland, OH, USA; 3 Dow University of Health and Sciences, Karachi, Pakistan

**Keywords:** allopurinol, granulomatous hepatitis, drug-induced liver injury

## Abstract

Liver enzyme elevation is a common reason for referral to a gastroenterologist. Drugs are one of the most common reasons for asymptomatic elevation of liver enzymes. We present here a case of granulomatous hepatitis (GH) secondary to long-term use of allopurinol. An 83-year-old male with a history of chronic gout and hypertension was evaluated for elevation of liver enzymes. He denies any complaints of abdominal pain, nausea, fever, chills, weight loss, night sweats, or yellowness of skin. He denies any use of herbal medications. He was on losartan and allopurinol for years. No new medications reported. Physical examination was unremarkable. Labs showed aspartate transaminase 101 U/L, alanine transaminase 81 U/L, and alkaline phosphatase 645 U/L. Ultrasound of the abdomen showed coarse liver texture. Liver biopsy was done that showed mixed GH. Given negative autoimmune and viral serologies, allopurinol-induced GH was suspected. Allopurinol was held, and repeat liver enzymes were checked in 3 months, which showed improvement in transaminase and alkaline phosphatase levels. This case highlights the importance of reviewing medications carefully when evaluating a patient with liver enzymes elevation, as stopping the offending drug can normalize the abnormalities in liver chemistries and can prevent subsequent expensive testing.

## Introduction

Hepatic granulomas (HGs) are not uncommon to find in liver biopsies. Of all liver biopsies done, granulomas are present in 2% to 10%, and of those, 13% to 36% are reported to have no definitive etiology even when evaluated extensively.^1,2^ HGs can be associated with a variety of diseases, including infections, autoimmune diseases, malignancies, and hypersensitivity reactions to drugs and chemical agents. The onset of HGs can be acute or chronic but acute onset is more likely drug-induced. HG may have no clinical manifestation and can be found incidentally. Most patients are asymptomatic. Symptoms including fever, rash, weight loss, anorexia, and night sweats may be present and usually indicate systemic disease. Sixty percent of cases are reported to have elevated liver enzymes on laboratory evaluation.^3^ Often, liver biopsy is required to establish the definitive diagnosis. Although rare, numerous drugs have been attributed to cause granulomatous hepatitis (GH), and allopurinol is one of the common causes.

Allopurinol is a xanthine oxidase inhibitor, a key enzyme in the uric acid cycle. It is the main therapeutic agent used to lower serum uric acid levels in patients with gout, cancer therapy–induced hyperuricemia, and also for recurrent calcium oxalate stones. Allopurinol, a simple and a cheaper drug, can be very effective if doses are adjusted adequately. Like many other drugs, it is associated with some adverse effects ranging from mild skin rashes to severe allopurinol-induced hypersensitivity. Here, we present a rare case of an 83-year-old male with chronic gout who developed allopurinol-induced granuloma.

## Case

**Figure 1.**
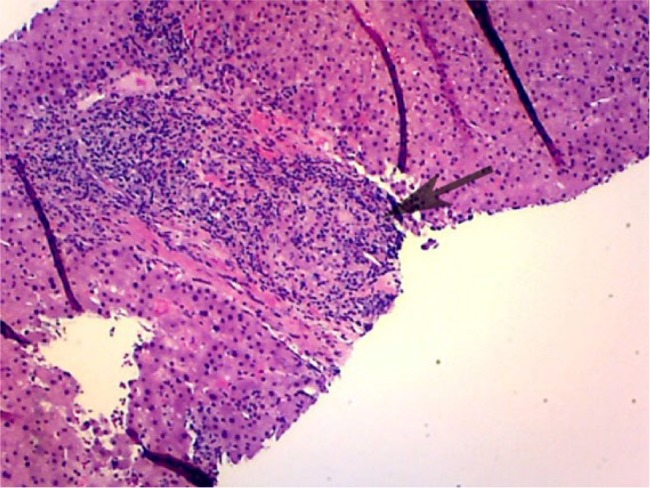
Well-formed epithelioid granuloma within one portal tract.

**Figure 2.**
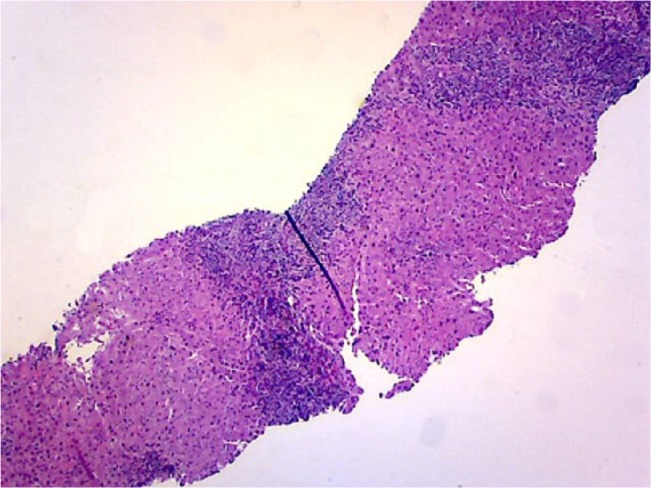
A low-power image showing the expanded portal tracts.

**Figure 3.**
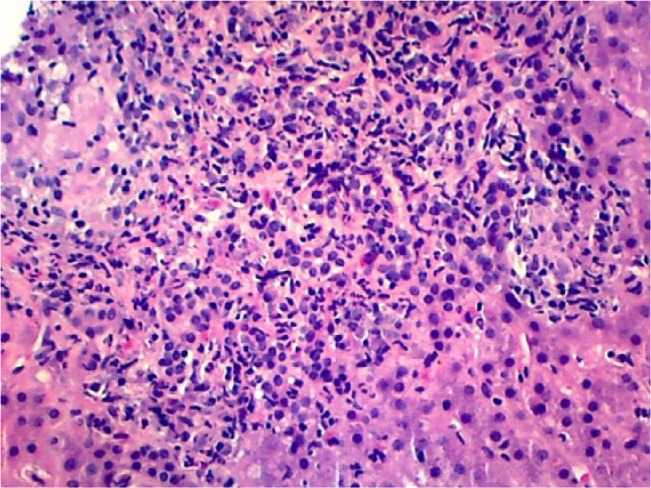
A higher power image showing the mixed nature of the inflammation (including lymphocytes, plasma cells, neutrophils, and eosinophils).

An 83-year-old male with a history of chronic gout and hypertension was evaluated for elevation of liver enzymes. He denies any complaints of abdominal pain, nausea, fever, chills, weight loss, night sweats, or yellowness of skin. He denies any use of herbal medications. He was a heavy drinker in the past but now has cut down his drinking to 2 beers a week. He was on losartan, statins, and allopurinol for years. No new medications were reported. Physical examination was unremarkable. Labs showed aspartate transaminase 101 U/L, alanine transaminase 81 U/L, and alkaline phosphatase 645 U/L. Hepatitis panel including hepatitis A, B, and C was unremarkable. Autoimmune workup including ANA and anti-smooth muscle antibody was unremarkable. Ultrasound of the abdomen showed coarse liver texture concerning for parenchymal disease. Liver biopsy was done, which showed mixed GH (Figures 1-3). Given negative autoimmune and viral serologies, allopurinol-induced GH was suspected. His liver chemistries improved after stopping allopurinol (Table 1).

**Table 1.** Trend of Liver Chemistries After Stopping Allopurinol.

	Reference Range	On Allopurinol	Three Months After Stopping Allopurinol
AST	17-59 U/L	101 (high)	57
ALT	21-72 U/L	81 (high)	44
ALP	38-125 U/L	645 (high)	306 (high)
Total bilirubin	0.0-1.0 mg/dL	0.7	0.5

Abbreviations: AST, aspartate transaminase; ALT, alanine transaminase; ALP, alkaline phosphatase.

## Discussion

The overall incidence of drug-induced HGs is thought to be 10%.^4^ Worldwide, infections are the most common cause of HG, but in developed countries sarcoidosis and primary biliary cirrhosis are thought to be more common.^5^ However, geographical location should also be considered while labeling etiology and frequency of disease. For example, in areas where parasitic infections are endemic, schistosomiasis should be kept in the differential diagnosis.^6^ Drugs are a common cause of GH. Several drugs are reported to cause GH, including antiseizure drugs (carbamezipine and phenytoin), antiparasitic drugs (mebendazole), calcium channel blockers (diltiazem), and sulfa drugs to name a few.

It may be challenging for a physician to label with certainty the relationship between HG and drug use. In our case, a male was presented with elevated liver enzymes, with a history of chronic gout and allopurinol use. All baseline workup was done to rule out other common etiologies, such as viral hepatitis and autoimmune diseases, which came out to be insignificant. A percutaneous liver biopsy was then performed, which showed HGs. Other common causes of HG, that is, sarcoidosis, tuberculosis, and primary biliary cirrhosis were ruled out. The suspicion was made that this granuloma could be due to allopurinol use. The diagnosis was supported by the improvement in the liver enzyme chemistry a few weeks after the withdrawal of the drug. A follow-up biopsy was offered but the patient declined.

The criteria used to diagnose drug-induced HG is based largely on ruling out other causes. Although biopsy plays a key role in diagnosing HGs, the morphology of drug-induced granulomas may be difficult to differentiate from other causes. A large number of eosinophils on biopsy may indicate a drug reaction, but parasites and Hodgkin’s disease must also be considered.^7^ A detailed and thorough history is helpful, including recent travel, occupational exposure of chemicals, and prescribed drugs with their dosages and duration of use. The latent period, which is the interval from the beginning of drug use till the onset of signs and symptoms, is also helpful and should be considered. For example, in the case of allopurinol, the latent period varies from 2 to 6 weeks.^8^

Once other causes of HGs are excluded and the diagnosis of drug-induced HG is established, the first step of management is to stop the offending drug. The injury caused by drug-induced HG is usually transient, with no significant complications.^9^ Treatment is mostly supportive with close monitoring of the clinical and laboratory course. Corticosteroids are not generally recommended for drug-induced liver injury and the use is limited to severe cholestatic jaundice with no improvement after removal of drug or concomitant extrahepatic manifestation secondary to hypersensitivity to the drug.^10^ Serial follow-up of liver chemistries should be done to evaluate for improvement. Patients with signs and symptoms of hepatic failure should be referred to a transplant center. Bilirubin levels >2 times along with alanine transaminase >3 times the upper normal limit are indicators of poor outcome following initiation of a drug, and should be evaluated by a hepatologist.^11^ A follow-up biopsy may be indicated to assess the resolution of granuloma, but it is not always necessary. In addition, a drug rechallenge test has almost never been done and is usually not recommended.

To make a clear temporal relationship between the drug and HG, great emphasis should be placed on a meticulous history. Allopurinol is a widely used drug in patients with gout. Fortunately, allopurinol-induced HG is uncommon. Even the resulting injury is transient and often resolves after discontinuation of the drug. Prevention is the key and can be done by patient education, appropriate dosing, and checking liver chemistries if liver dysfunction is suspected.

The unique aspect of this case is the development of liver abnormalities many years (>5 years) after being on allopurinol, which is unusual as we usually expect liver abnormalities to appear within days to weeks of starting treatment. So clinicians should not exclude the possibility of GH secondary to allopurinol in patients on chronic therapy with it as liver abnormalities can develop many years after treatment.

## References

[1] Harrington PT Gutiérrez JJ Ramirez-Ronda CH Quiñones-Soto R Bermúdez RH Chaffey J Granulomatous hepatitis. Rev Infect Dis. 1982;4:638-655. 7123041 10.1093/clinids/4.3.638

[2] Lamps LW Hepatic granulomas: a review with emphasis on infectious causes. Arch Pathol Lab Med. 2015;139:867-875. 26125427 10.5858/arpa.2014-0123-RA

[3] Flamm SL Granulomatous liver disease. Clin Liver Dis. 2012;16:387-396. 22541705 10.1016/j.cld.2012.03.013

[4] Kleiner DE Granulomas in the liver. Semin Diagn Pathol. 2006;23:161-169. 17355089 10.1053/j.semdp.2006.11.003

[5] McCluggage WG Sloan JM Hepatic granulomas in Northern Ireland: a thirteen year review. Histopathology. 1994;25:219-228. 7821889 10.1111/j.1365-2559.1994.tb01321.x

[6] Matheus T Muñoz S Granulomatous liver disease and cholestasis. Clin Liver Dis. 2004;8:229-246. 15062203 10.1016/S1089-3261(03)00137-5

[7] Mills PR Russell RI Diagnosis of hepatic granulomas: a review. J R Soc Med. 1983;76:393-397. 6345773 10.1177/014107688307600513 PMC1439165

[8] Gaya DR Thorburn D Oien KA Morris AJ Stanley AJ Hepatic granulomas: a 10 year single centre experience. J Clin Pathol. 2003;56:850-853. 14600131 10.1136/jcp.56.11.850 PMC1770104

[9] Katsuma A Shibata M Katsuki T Imai E Tada M Hinoshita F A case of acute interstitial nephritis and granulomatous hepatitis induced by ingesting quinine. CEN Case Rep. 2015;4:76-80. 28509275 10.1007/s13730-014-0143-0 PMC5413713

[10] Reuben A Hy’s law. Hepatology. 2004;39:574-578. 14768020 10.1002/hep.20081

[11] Giannattasio A D’Ambrosi M Volpicelli M Iorio R Steroid therapy for a case of severe drug-induced cholestasis. Ann Pharmacother. 2006;40:1196-1199. 16720710 10.1345/aph.1G345

